# Different Ectopic Hoxa2 Expression Levels in Mouse Cranial Neural Crest Cells Result in Distinct Craniofacial Anomalies and Homeotic Phenotypes

**DOI:** 10.3390/jdb10010009

**Published:** 2022-01-31

**Authors:** Taro Kitazawa, Maryline Minoux, Sebastien Ducret, Filippo M. Rijli

**Affiliations:** 1 Friedrich Miescher Institute for Biomedical Research, Maulbeerstrasse 66, 4058 Basel, Switzerland; taro.kitazawa@fmi.ch (T.K.); m.minoux@unistra.fr (M.M.); sebastien.ducret@fmi.ch (S.D.); 2 INSERM UMR 1121, Faculté de Chirurgie Dentaire, Université de Strasbourg, 8 rue Sainte Elisabeth, 67 000 Strasbourg, France; 3 Departement Biozentrum, University of Basel, Spitalstrasse 41, 4056 Basel, Switzerland

**Keywords:** *Hox* genes, Hoxa2, Hoxa5, cranial neural crest cells, gain-of-function, craniofacial morphogenesis, pinna, middle ear, hyoid, homeotic transformation, hypoplasia

## Abstract

Providing appropriate positional identity and patterning information to distinct rostrocaudal subpopulations of cranial neural crest cells (CNCCs) is central to vertebrate craniofacial morphogenesis. *Hox* genes are not expressed in frontonasal and first pharyngeal arch (PA1) CNCCs, whereas a single *Hox* gene, *Hoxa2*, is necessary to provide patterning information to second pharyngeal arch (PA2) CNCCs. In frog, chick and mouse embryos, ectopic expression of *Hoxa2* in *Hox*-negative CNCCs induced hypoplastic phenotypes of CNCC derivatives of variable severity, associated or not with homeotic transformation of a subset of PA1 structures into a PA2-like identity. Whether these different morphological outcomes are directly related to distinct *Hoxa2* overexpression levels is unknown. To address this issue, we selectively induced *Hoxa2* overexpression in mouse CNCCs, using a panel of mouse lines expressing different *Hoxa2* ectopic expression levels, including a newly generated *Hoxa2* knocked-in mouse line. While ectopic *Hoxa2* expression at only 60% of its physiological levels was sufficient for pinna duplication, ectopic *Hoxa2* expression at 100% of its normal level was required for complete homeotic repatterning of a subset of PA1 skeletal elements into a duplicated set of PA2-like elements. On the other hand, ectopic *Hoxa2* overexpression at non-physiological levels (200% of normal levels) led to an almost complete loss of craniofacial skeletal structures. Moreover, ectopic *Hoxa5* overexpression in CNCCs, while also resulting in severe craniofacial defects, did not induce homeotic changes of PA1-derived CNCCs, indicating *Hoxa2* specificity in repatterning a subset of *Hox*-negative CNCCs. These results reconcile some discrepancies in previously published experiments and indicate that distinct subpopulations of CNCCs are differentially sensitive to ectopic levels of *Hox* expression.

## 1. Introduction

The multipotent neural crest cells delaminate dorsally along the rostrocaudal axis of the forming neural tube and migrate towards various regions of the developing embryo, where they locally differentiate into a broad range of cell lineages [1]. In the head, cranial neural crest cells (CNCCs) colonize the frontonasal process (FNP) and pharyngeal arches (PAs), providing the main source of skeletogenic head mesenchyme, central to craniofacial and pharyngeal morphogenesis. Rostral CNCCs give rise to the frontonasal skeleton and make extensive contributions to the membranous bones of the skull. More posterior CNCCs fill the PAs, forming the cartilages and bones of the upper and lower jaws, middle ear ossicles, outer ear, hyoid and thyroid structures [1,2,3,4]. Defects of regional specification and patterning of different CNCC subpopulations lead to distinct craniofacial abnormalities [5].

Rostrocaudally, a fundamental molecular difference among CNCC subpopulations concerns their patterns of *Hox* gene expression. CNCCs contributing to the FNP and first pharyngeal arch (PA1) do not express *Hox* genes, whereas CNCCs contributing to the second and more posterior arches display nested combinatorial *Hox* expression patterns, providing each PA with distinct regional identities along the rostrocaudal axis [3,4,6,7,8]. The role of *Hox* genes in establishing rostrocaudal identity of CNCC derivatives first became evident with the targeted inactivation of *Hoxa2* in the mouse, which resulted in homeotic transformation of PA2 into a subset of PA1-like derivatives [9,10]. Namely, in *Hoxa2* knockout mice, the PA2-derived stapes, styloid process and lesser horns of hyoid bone were absent and replaced by proximal PA1-like structures including the malleus, incus, gonial bone and tympanic ring in a mirror image pattern of their normal counterparts [9,10]. Furthermore, the outer ear or pinna, a PA2-derived structure [11], was absent as well and replaced by a duplication of the PA1-derived external auditory canal [10,11,12]. *Hoxa2* downregulation in *Xenopus* [13] and zebrafish [14] also induced PA2 to PA1-like homeotic transformation of skeletal elements, indicating an essential and conserved role of *Hoxa2* for PA2 CNCC identity specification.

In contrast, distinct experiments analyzing *Hoxa2* ectopic expression in *Hox*-negative CNCCs led to different morphological phenotypes. Several studies reported a repatterning of proximal PA1 into PA2-like derivatives [11,14,15,16,17], while this phenotype was not observed by others [18]. Rather, it was reported that forced expression of *Hoxa2* in chick *Hox*-negative CNCCs abolishes the development of the entire facial skeleton, including frontonasal structures and jaws [18]. These dramatic phenotypes might be caused by abnormal persistent expression of *Hoxa2* in the chondrogenic regions [19]. To explain these discrepancies, it was initially proposed that a morphological transformation could only be obtained following global overexpression of *Hoxa2*, both in the CNCCs and surrounding tissues. When only CNCCs were targeted in chick embryos, the loss of facial structures was observed but not homeotic repatterning of PA1 elements [15,18].

However, recent studies in the mouse have shown that *Wnt1-Cre*-dependent conditional ectopic expression of *Hoxa2* in CNCCs is alone sufficient to induce a repatterning of proximal PA1 into PA2-like skeletal elements, including mirror image duplication of the pinna [11,16]. Notably, the development of more anterior skeletal structures, including frontonasal elements and jaws, was impaired as well [11,16]. Namely, short snout and exencephaly were observed, and skull vault (frontal), facial (nasal, premaxillary), skull base (nasal septum, vomer, palatine, pterygoid, alisphenoid), lower jaw (dentary) and upper jaw (maxillary, jugal, squamous) skeletal elements became hypoplastic and malformed. These findings indicated a differential ability of *Hoxa2* to repattern *Hox*-negative CNCC subpopulations [11,16].

Lastly, little is known about the specificity of *Hox* genes in the repatterning of *Hox*-negative CNCC derivatives. In the chick, Creuzet et al., 2002 [18], showed that ectopic expression of *Hoxa2*, *Hoxa3* or *Hoxb4* in *Hox*-negative CNCCs had negative effects on facial skeleton formation, albeit at different extents. While ectopic *Hoxa2* expression caused the absence of the entire *Hox*-negative CNCC derivatives, the effects of *Hoxa3* and *Hoxb4* were more restricted [18]. However, since Creuzet et al., 2002 [18], did not observe homeotic repatterning of a subset of *Hox*-negative CNCCs following *Hoxa2* overexpression, it is still unclear whether ectopic expression of *Hox* genes other than *Hoxa2* in the mouse might also induce repatterning into PA2-like derivatives.

One possibility to reconcile the distinct morphological phenotypes reported by the different studies is that different threshold levels of ectopic *Hoxa2* expression may differentially affect the morphogenesis of *Hox*-negative CNCC subpopulations. Here, we evaluated how distinct ectopic *Hoxa2* expression levels differentially affect *Hox*-negative CNCC subpopulation morphogenesis. To this aim, we induced *Wnt1::Cre*-dependent conditional *Hoxa2* overexpression in CNCCs using mouse lines that generate different *Hoxa2* ectopic expression levels. Namely, we used our previously reported mouse allele [11] and showed that it achieves *Hoxa2* ectopic expression at only 60% of endogenous levels in PA2, and a newly generated allele, which expresses high, non-physiological, levels of ectopic *Hoxa2*, namely 200% of endogenous levels. We further compared the obtained phenotypes with those described in Kitazawa et al., 2015 [16], which achieved *Wnt1::Cre*-dependent conditional ectopic *Hoxa2* expression in *Hox*-negative CNCCs comparable to physiological endogenous levels (100%) in PA2. In this study, a repatterning of proximal PA1 into a full set of PA2-like derivatives (skeletal elements and pinna) was observed alongside with a severe impairment of more anterior skeletal structures [16]. Here, we found that *Hoxa2* overexpression at 60% of its physiological level induced only a partial repatterning of proximal PA1 into PA2-like skeletal elements, including full duplication of the pinna, and relatively modest hypoplasia of more anterior skeletal structures. In contrast, high, non-physiological, ectopic levels (200%) of *Hoxa2* overexpression caused dramatic hypoplasia and/or aplasia of craniofacial elements including PA2 and proximal PA1 derivatives, without clear homeotic phenotype. Lastly, we assessed *Hox* gene specificity in repatterning of craniofacial elements by analyzing mice displaying *Wnt1::Cre*-dependent *Hoxa5* conditional overexpression in CNCCs. We found that overexpression of *Hoxa5* in CNCCs induced a loss of proximal PA1 and PA2–PA4 structures without homeotic transformation, indicating specificity of *Hoxa2* in the repatterning of a subset of PA1 into PA2-like CNCC derivatives.

## 2. Materials and Methods

### 2.1. Animals

All animal experiments were performed in accordance with Guide for Care and Use of Laboratory Animals and were approved by the Veterinary Department of the Canton of Basel-Stadt.

### 2.2. Generation of the ROSA26^CAG::(lox-stop-lox)-Hoxa2-IRES-EGFP-WPRE^ Mouse Line

We generated a mouse line that utilizes *CAG* promoter and *WPRE* sequence to induce high levels of *Hoxa2* overexpression (*ROSA26^CAG::(lox-stop-lox)-Hoxa2-IRES-EGFP-WPRE^*) in a *Cre*-dependent manner, with a similar strategy as for the *ROSA26^CAG::(lox-stop-lox)-Hoxa5-IRES-EGFP-WPRE^* mouse line [20]. By homologous recombination, we introduced in the *ROSA26* locus the targeting vector pR26-CAG-lsl-3xFLAG-Hoxa2-IRES-GFP, consisting of a *CAG* promoter, a lox-stop-lox cassette, a *Hoxa2* tagged with a *3xFLAG*, an *IRES-GFP*, a *WPRE* element, a *bGH poly(A)* and a *PGK-Neo* cassette. To generate this plasmid, we used pR26-CAG-lsl-Kir (kind gift from Guillermina López-Bendito; [21]), in which we replaced the insert located between the two FseI restriction sites by the cassette 3xFLAG-Hoxa2-IRES-GFP (PCR amplified from the plasmid pROSA26-FLAG-Hoxa2-IRES-EGFP [22] and cloned into the TOPO vector pCRII (Invitrogen) with insertion of a 3xFLAG tag). This vector was linearized with PvuI and electroporated into the E14 embryonic stem cell (ESC) line. After G418 resistance-selection and screening by PCR, positive ESC clones were aggregated with morula-stage embryos obtained from inbred (C57BL/6 X DBA/2) F1 mice. Germline transmission of the *ROSA26^CAG::(lox-stop-lox)-Hoxa2-IRES-EGFP-WPRE^* allele was obtained. Heterozygous and homozygous mice were viable and fertile.

### 2.3. Mouse Lines and Mating Scheme

*ROSA26^(lox-stop-lox)-Hoxa2-IRES-EGFP^* [22], *ROSA26^CAG::(lox-stop-lox)-Hoxa5-IRES-EGFP-WPRE^* [20] and newly generated *ROSA26^CAG::(lox-stop-lox)-Hoxa2-IRES-EGFP-WPRE^* alleles were initially generated in the background of E14 ESCs. Germline-transmitted alleles were crossed with the CD1 mouse line and maintained as homozygous mice. Homozygous *ROSA26^(lox-stop-lox)-Hoxa2-IRES-EGFP^*, *ROSA26^CAG::(lox-stop-lox)-Hoxa2-IRES-EGFP-WPRE^* or *ROSA26^CAG::(lox-stop-lox)-Hoxa5-IRES-EGFP-WPRE^* females were crossed with the *Wnt1::Cre* transgenic mouse line [23] on C57B/6 background (JAX stock #02250) to generate *Wnt1::Cre;ROSA26^(lox-stop-lox)-Hoxa2-IRES-EGFP^* (*Wnt1-Hoxa2-low*), *Wnt1::Cre; ROSA26^CAG::(lox-stop-lox)-Hoxa2-IRES-EGFP-WPRE^* (*Wnt1-Hoxa2-high*) or *Wnt1::Cre;ROSA26^CAG::(lox-stop-lox)-Hoxa5-IRES-EGFP-WPRE^* (*Wnt1-Hoxa5*) mice, respectively.

### 2.4. Skeletal Staining

Skeletal staining of mouse E18.5 *Wnt1-Hoxa2-low* (*n* = 4), *Wnt1-Hoxa2-high* (*n* = 6) and *Wnt1-Hoxa5* (*n* = 9) fetuses was performed according to a previously described protocol with minor modifications [16,24]. Additionally, we re-analyzed E18.5 skeletons from our previous study [11] generated by crossing *ROSA26 ^(lox-stop-lox)-Hoxa2-IRES-EGFP^* mice with a distinct *Wnt1::Cre* line [25] (*n* = 6), showing undistinguishable phenotype from the *Wnt1-Hoxa2-low* mice reported here. Samples were fixed in 95% ethanol for 5–7 days. Subsequently, embryos were incubated with 0.015% alcian blue 8GS, 0.005% alizarin red S and 5% acetic acid for 3 days with agitation at 37 °C. Samples were cleared in 1% KOH for several days and in 1% KOH/glycerol series until surround tissues turned transparent. The samples were stored in glycerol for a longer term.

### 2.5. RT-qPCR Analysis of mRNA Expression Levels

We carried out RNA extraction from PA1 and PA2 in E10.5 wild-type, *Wnt1-Hoxa2-low* and *Wnt1-Hoxa2-high* mice in a comparable manner with previous *Wnt1-Hoxa2-medium* mice analysis [16]. The PA1 mandibular and PA2 were manually dissected from E10.5 embryos, and 2–3 pairs of PAs were pooled. Subsequently, total RNA was purified by RNeasy Mini Kit (Qiagen, Germantown, MD 20874, USA, #74104) with genomic DNA digestion using RNase-Free DNase I Set (Qiagen, Germantown, MD, USA, #79254) according to the manufactures’ protocol. 1 μg of total RNA was reverse-transcribed using Superscript III Reverse Transcriptase (Thermo Fisher, Waltham, MA, USA, #18080093) and oligo(dT) primer (Thermo Fisher, Waltham, MA, USA, #SO131). Quantification of mRNA was performed by real-time PCR using StepOnePlus Real-Time PCR System with SYBR Green PCR Master Mix. Quantification of *Hoxa2* and *Gapdh* was performed by ΔΔCt using *Gapdh* as an internal control. Statistical analysis was performed by one-way analysis of variance (ANOVA) followed by Tukey’s honest significant difference (HSD) post hoc tests.

## 3. Results

### 3.1. Analysis of Mouse Phenotypes Induced by Low Levels of Conditional Ectopic Hoxa2 Expression in CNCCs

In a previous study [16], *Wnt1::Cre* [25]-dependent conditional ectopic *Hoxa2* overexpression was achieved in mouse *Hox*-negative premigratory CNCC progenitors by knock-in of a *CAG promoter-(lox-stop-lox)-Hoxa2* cassette into *ROSA26* to generate the *ROSA^CAG::(lox-stop-lox)-Hoxa2^* mouse line. RT-qPCR analysis in E10.5 embryos showed that ectopic *Hoxa2* expression in PA1 was at a level comparable to endogenous *Hoxa2* expression in wild-type PA2 (100%) [16]. We refer, hereafter, to this line as *Wnt1-Hoxa2-medium*.

In *Wnt1-Hoxa2-medium* fetuses, ectopic *Hoxa2* expression resulted in two categories of phenotypes ([16]; final summary schemes of this study). The proximal part of PA1 was homeotically transformed into PA2-like skeletal elements. Namely, malleus, incus, tympanic ring and gonial elements were repatterned into ectopic lesser horn and body of hyoid bone, styloid process and stapes elements in a mirror image pattern of their normal counterparts, with a slight hypoplasia. *Wnt1-Hoxa2-medium* fetuses also displayed a duplication of the pinna, even though both normal and ectopic pinnae were mildly hypoplastic as compared to wild-type pinna. Moreover, the development of the other *Hox*-negative CNCC subpopulations, including the FNP and distal part of PA1, was severely impaired.

**Figure 1:**
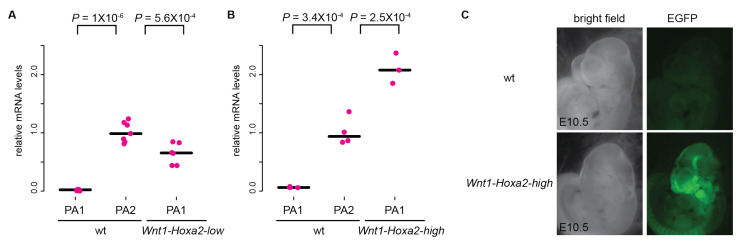
Ectopic *Hoxa2* expression in *Wnt1-Hoxa2-low* and *Wnt1-Hoxa2-high* mouse embryos. (**A**,**B**) Quantification of ectopic *Hoxa2* mRNA expression levels in E10.5 *Wnt1-Hoxa2-low* (**A**) and *Wnt1-Hoxa2-high* (**B**) embryos by RT-qPCR. *Hoxa2* mRNA levels were quantified in PA2 and PA1 of wild-type (wt) embryos and in PA1 of *Hoxa2* overexpressing embryos. *Hoxa2* mRNA levels were normalized by comparing with *Gapdh* mRNA. Data are from *n* = 7 (wt in **A**), *n* = 6 (*Wnt1-Hoxa2-low* in **A**), *n* = 4 (wt in **B**) and *n* = 3 (*Wnt1-Hoxa2-high* in **B**) biologically independent samples. *p*-values are from analysis of variance followed by Tukey’s HAD post hoc tests, and bars indicate the median. (**C**) E10.5 wt (top) and *Wnt1-Hoxa2-high* (bottom) embryos in bright field (left) and EGFP (right) signals. PA, pharyngeal arch.

To assess how differential ectopic *Hoxa2* expression levels affect *Hox*-negative CNCCs morphogenesis, we firstly reinvestigated our previous *ROSA^(lox-stop-lox)-Hoxa2-IRES-EGFP^* mouse line [11,22], where a (*lox-stop-lox)-Hoxa2-IRES-EGFP* cassette was knocked-in to the *ROSA26* locus. To conditionally overexpress *Hoxa2* in CNCCs, this line was crossed with the *Wnt1::Cre* transgenic mouse line [23]. RT-qPCR analysis of PA1 mRNA in E10.5 embryos revealed that the ectopic *Hoxa*2 expression driven by the *ROSA26* promoter was significantly lower, about 60%, as compared to the endogenous *Hoxa2* expression in wild-type PA2 (Figure 1A), likely because the *ROSA26* promoter is weaker than the *CAG* promoter in Kitazawa et al., 2015 [16]. We refer, hereafter, to the *Wnt1::Cre; ROSA ^(lox-stop-lox)-Hoxa2-IRES-EGFP^* line as *Wnt1-Hoxa2-low*. We also re-analyzed *ROSA26^(lox-stop-lox)-Hoxa2-IRES-EGFP^* mice from our previous study [11] crossed with a distinct *Wnt1::Cre* line [25], showing undistinguishable phenotype from *Wnt1-Hoxa2-low* specimen (see below).

**Figure 2:**
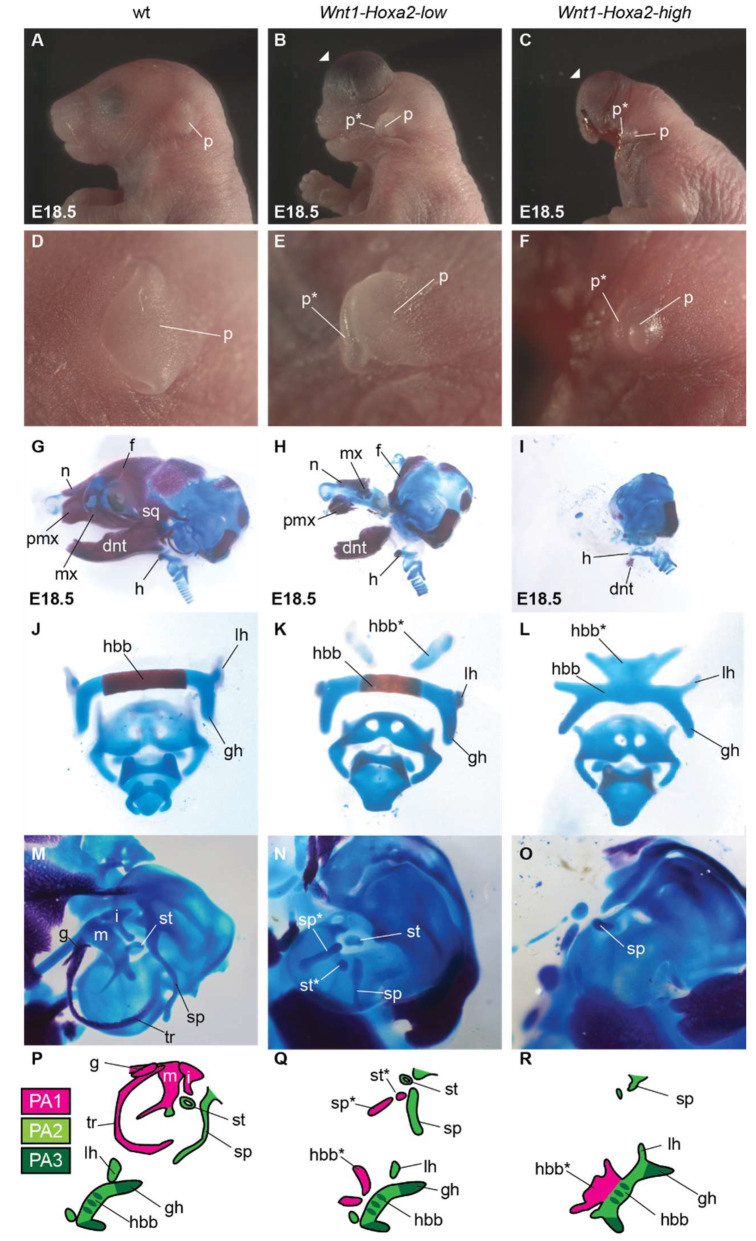
Craniofacial phenotypes of *Wnt1-Hoxa2-low* and *Wnt1-Hoxa2-high* mouse fetuses. E18.5 wild-type (wt, **A**,**D**,**G**,**J**,**M**,**P**), *Wnt1-Hoxa2-low* (**B**,**E**,**H**,**K**,**N**,**Q**) and *Wnt1-Hoxa2-high* (**C**,**F**,**I**,**L**,**O**,**R**) fetuses are compared. (**A**–**C**) Lateral views of facial appearance. Short snout and exencephaly (arrowheads) are more severe in *Wnt1-Hoxa2-high* fetuses. (**D**–**F**) Higher magnification of the pinna. Duplication of the pinna is observed in both *Wnt1-Hoxa2-low* (**E**) and *Wnt1-Hoxa2-high* (**F**) specimen; in (**F**), normal and ectopic pinnae are hypoplastic. (**G**–**I**) Lateral views of head and neck skeletal staining. In (**I**), structures are absent or hypoplastic, more than in (**H**). (**J**–**L**) Higher magnification of the hyoid region. In *Wnt1-Hoxa2-low* (**K**), the ectopic body of the hyoid bone (hbb*) is present in only 70% of the mutant fetuses. In *Wnt1-Hoxa2-high* (**L**), a malformed hbb* is always present. (**M**–**R**) Higher magnification of the middle ear and hyoid regions (**M**–**O**) and their schematic representations (**P**–**R**). Skeletal elements derived from PA1 (magenta), PA2 (light green) and PA3 (dark green) are labelled. dnt, dentary; f, frontal; g, gonial; gh, greater horn of hyoid bone; h, hyoid bone; hbb, body of hyoid bone; i, incus; lh, lesser horn of hyoid bone; m, malleus; mx, maxillary; n, nasal; p, pinna; pmx, premaxillary; PA, pharyngeal arch; sp, styloid process; sq, squamosal; st, stapes; tr, tympanic ring; *, duplicated structure.

*Wnt1-Hoxa2-low* E18.5 fetuses displayed a fully duplicated pinna, as previously reported [11] (Figure 2A–E). They also display a duplication of the styloid process and stapes elements in a mirror image pattern of their normal counterparts (Figure 2N,Q; final summary schemes ), even though both the ectopic and normal elements were hypoplastic. However, repatterning of the other proximal PA1 skeletal structures into PA2-like morphology was incomplete (Figure 2J,K,M,N,P,Q). An ectopic body of hyoid bone was observed in only 70% (7/10) of samples (Figure 2K,N,Q) and duplication of the lesser horn of hyoid bone was never observed (Figure 2K,N,Q). Thus, lower than physiological levels of ectopic *Hoxa2* expression can induce pinna duplication, while not sufficient for the complete homeotic transformation of skeletal elements. Moreover, in E18.5 *Wnt1-Hoxa2-low* fetuses, structures derived from the FNP and distal PA1, e.g., facial, nasal, premaxillary, nasal septum, vomer, palatine, pterygoid, alisphenoid, dentary, maxillary, jugal and squamous, were reduced or absent. This suggests that the severity of hypoplasia and malformation or aplasia of individual elements derived from rostral *Hox*-negative CNCC subpopulations is differentially dependent on local levels of ectopic *Hoxa2* expression.

### 3.2. Analysis of Mouse Phenotypes Induced by High Levels of Conditional Ectopic Hoxa2 Expression in CNCCs

To assess how high, non-physiological, levels of *Hoxa2* overexpression affect the morphogenesis of *Hox*-negative CNCCs, we generated a novel *Hoxa2* overexpression mouse line by knocking-in a *CAG promoter-(lox-stop-lox)-Hoxa2-IRES-EGFP-woodchuck hepatitis virus posttranscriptional regulatory element (WPRE)* cassette into the mouse *ROSA26* locus to generate *ROSA^CAG::(lox-stop-lox)-Hoxa2-IRES-EGFP-WPRE^* mice. The *CAG* promoter is shared with the *ROSA^CAG::(lox-stop-lox)-Hoxa2^* mouse line [16], but the *WPRE* sequence further enhances the expression of the transgene by increasing nuclear and cytoplasmic mRNA transcript levels [26,27]. To overexpress *Hoxa2* in CNCCs, we crossed this line with the *Wnt1::Cre* transgenic mouse line [23]. In E10.5 embryos, we confirmed expression of EGFP in CNCCs, allowed by the *IRES* sequence (Figure 1C). In addition, RT-qPCR analysis of *Hoxa2* mRNA in PA1 revealed that its ectopic expression level was significantly higher, about 200%, as compared with wild-type PA2 endogenous *Hoxa2* expression (Figure 1B). We refer, hereafter, to the *Wnt1::Cre; ROSA^CAG::(lox-stop-lox)-Hoxa2-IRES-EGFP-WPRE^* line as *Wnt1-Hoxa2-high*.

E18.5 *Wnt1-Hoxa2-high* fetuses showed extensive hypoplasia and/or aplasia of craniofacial structures derived from FNP and distal PA1 (Figure 2C,I; final summary schemes). In particular, most of the lower jaw (dentary bone) and nasal septum were largely absent and replaced by several cartilage nodules (Figure 2I), which might include mesoderm-derived hypochiasmatic cartilage [28]. In addition, E18.5 *Wnt1-Hoxa2-high* fetuses showed strong hypoplasia and malformation of the craniofacial elements derived from the PA2 and proximal PA1 CNCCs (Figure 2F,L,O,R; final summary schemes). Consequently, no homeotic transformation could be observed in the middle ear: the malleus, incus, gonial, tympanic ring and stapes elements were absent, and the styloid process was strongly malformed (Figure 2O,R). In the hyoid region, an ectopic body of hyoid bone was present but strongly malformed, and an ectopic lesser horn of hyoid bone was not observed (Figure 2L,R). Moreover, the PA2-derived body and lesser horns of hyoid bone were malformed and fused (Figure 2L,R). While we could still observe duplication of the pinna, both PA1- and PA2-derived pinnae were much smaller than wild-type pinna (Figure 2F).

In summary, malformation or absence of craniofacial structures was evidently more severe in *Wnt1-Hoxa2-high* mice as compared with *Wnt1-Hoxa2-low* and *Wnt1-Hoxa2-medium* mice ([16]; Figure 2; final summary schemes), with no clear homeotic phenotype (except for pinna duplication—a structure lacking in chick embryos), thus mimicking the dramatically hypoplastic craniofacial phenotypes of *Hoxa2*-overexpressing chick embryos in Creuzet et al., 2002 [18].

### 3.3. Analysis of Mouse Phenotypes Induced by Conditional Ectopic Hoxa5 Overexpression in CNCCs

To assess the specificity of ectopically-expressed *Hoxa2* in the repatterning of CNCC derivatives, we induced conditional overexpression in CNCCs of *Hoxa5*, a *Hox* gene not expressed in neural crest cells contributing to craniofacial structures. To this aim, similar to the construction of the *Wnt1-Hoxa2-high* allele, a *CAG promoter-(lox-stop-lox)-Hoxa5-IRES-EGFP-WPRE* cassette, driving high levels of *Hoxa5* expression, was knocked-in to the *ROSA26* locus to generate the *ROSA^CAG::(lox-stop-lox)-Hoxa5-IRES-EGFP-WPRE^* mouse line [20]. This line was crossed with the *Wnt1::Cre* transgenic mouse line [23]. We refer, hereafter, to the *Wnt1::Cre*; *ROSA^CAG::(lox-stop-lox)-Hoxa5-IRES-EGFP-WPRE^* line as *Wnt1-Hoxa5.*

**Figure 3:**
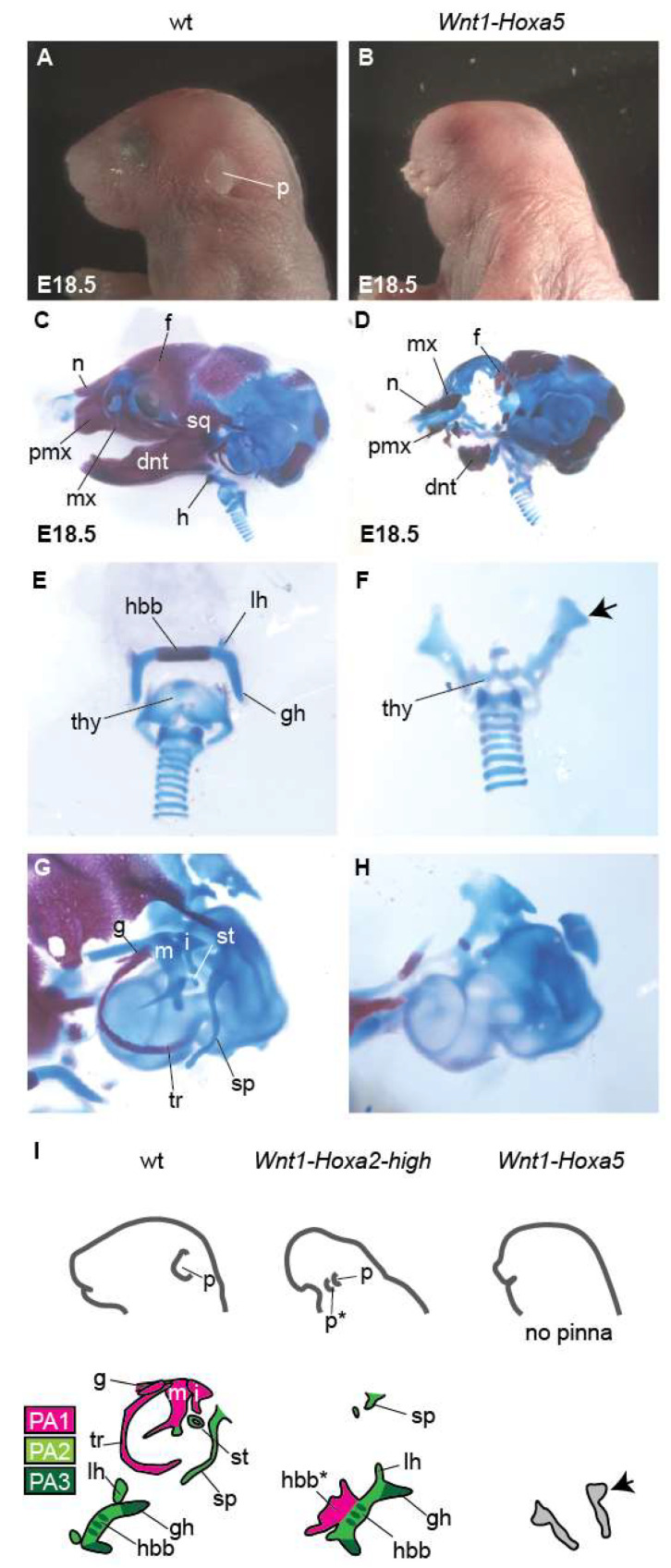
Craniofacial phenotype of *Wnt1-Hoxa5* mouse fetuses. E18.5 wild-type (wt, **A, C, E, G**) and *Wnt1-Hoxa5* (**B**,**D**,**F**,**H**) fetuses were compared. (**A**,**B**) Lateral views of external appearance. The pinna (p) is absent in *Wnt1-Hoxa5* fetuses. (**C**,**D**) Lateral views of skeletal staining. In (**D**), *Wnt1-Hoxa5* hypoplastic phenotype is less severe than in *Wnt1-Hoxa2-high* fetuses (Figure 2I). (**E**,**F**) Higher magnification of the hyoid region. In (**F**), hyoid elements are absent and thyroid cartilage (thy) is reduced; (arrow) ectopic cartilage. (**G**,**H**) Higher magnification of middle ear structures. In (**H**), middle ear structures are absent. (**I**) Schematic representations of pinna (upper row) and middle ear and hyoid (lower row) phenotypes in *Wnt1-Hoxa2-high* (see also Figure 2) and *Wnt1-Hoxa5* fetuses; (arrow), ectopic cartilage in hyoid region: note that it is difficult to assign its regional origin. dnt, dentary; f, frontal; g, gonial; gh, greater horn of hyoid bone; h, hyoid bone; hbb, body of hyoid bone; i, incus; lh, lesser horn of hyoid bone; m, malleus; mx, maxillary; n, nasal; p, pinna; pmx, premaxillary; sp, styloid process; sq, squamosal; st, stapes; thy, thyroid; tr, tympanic ring; *, duplicated structure.

As a proxy for *Hoxa5* overexpression from the *Hoxa5-IRES-EGFP-WPRE* allele, we detected EGFP expression at E18.5 (Figure S1). E18.5 *Wnt1-Hoxa5* fetuses showed hypoplasia and malformation of craniofacial structures derived from FNP and distal PA1 CNCCs, albeit milder as compared with *Wnt1-Hoxa2-high* fetuses (Figure 2 and Figure 3A–D). In particular, the short snout and exencephaly were less severe. In addition, many skeletal elements including facial, nasal, premaxillary, nasal septum, alisphenoid, dentary and maxillary elements were reduced but still present in *Wnt1-Hoxa5* fetuses (Figure 3A–D).

On the other hand, in E18.5 *Wnt1-Hoxa5* fetuses, PA2 and proximal PA1-derived structures were severely affected. The pinna was absent (Figure 3A,B,I). Furthermore, hyoid bone (body, greater and lesser horn) and middle ear elements (malleus, incus, gonial bone, tympanic ring, stapes and styloid process) were absent, without homeotic transformation into PA2-like derivatives (Figure 3E–I). In the hyoid region, ectopic rod-like cartilage nodules were formed (Figure 3F,I, arrows). In addition, PA4-derived thyroid cartilage, which was not affected by *Hoxa2* overexpression (Figure 2J–L), was hypoplastic (Figure 3E,F).

In summary, *Hoxa5* ectopic expression in CNCCs caused more severe hypoplasia and malformation of proximal PA1 and PA2–PA4 than FNP and distal PA1 CNCC derivatives and could not induce homeotic transformation of PA1 into PA2-like elements. These results indicate a specificity of *Hoxa2* in the ability to selectively repattern proximal PA1 elements.

## 4. Discussion

### 4.1. Homeotic Repatterning of Hox-Negative CNCCs by Hoxa2 is Dependent on Its Ectopic Expression Levels and CNCC Position within the Craniofacial Complex

In mouse PA2 CNCCs, *Hoxa2* regulates the spatial distribution of chondrocytes and inhibits intra-membranous ossification [19,29], providing critical patterning information for morphogenesis [9,10]. The effects of ectopic *Hoxa2* expression on *Hox*-negative CNCCs have been analyzed in different vertebrate species. Several studies have reported homeotic transformation of proximal PA1 to PA2-like derivatives [11,14,15,17,24]. In contrast, in other studies, ectopic *Hoxa2* expression in CNCCs resulted in severely impaired skull and facial development, without homeotic transformation [15,18,30]. On the other hand, inhibitory effects of *Hoxa2* overexpression on chondrogenesis and osteogenesis have been reported when *Hoxa2* expression was constitutively forced in *Col2a1*-expressing developing chondrocytes [29,31].

**Figure 4:**
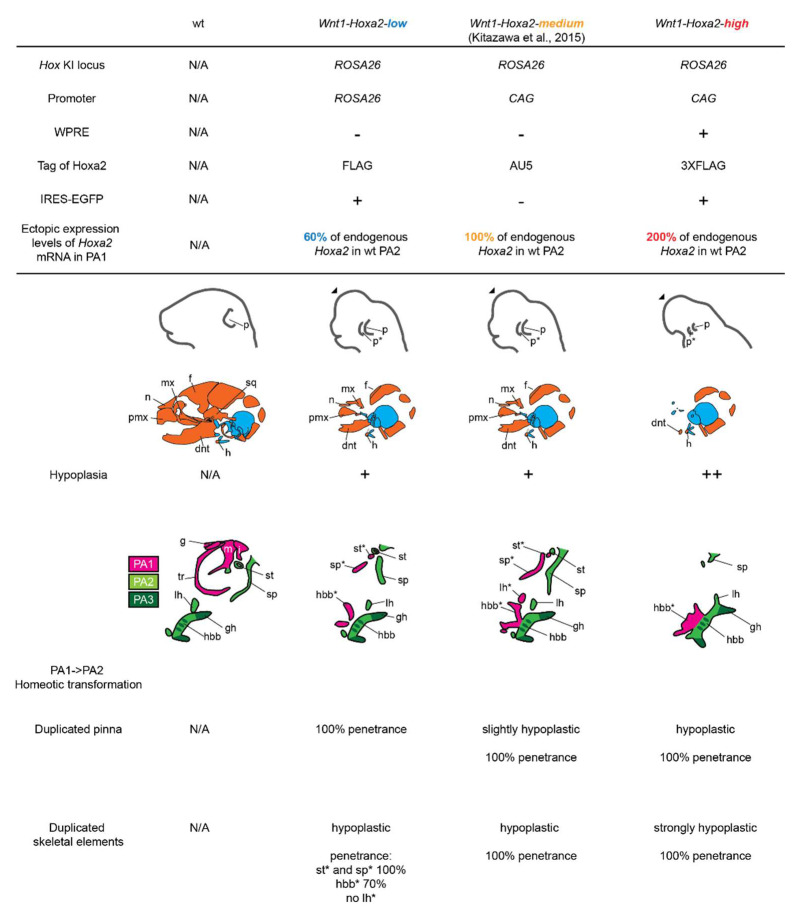
Craniofacial phenotypes induced by distinct ectopic *Hoxa2* expression levels. (Upper rows) Design of the *Wnt1::Cre*-dependent conditional *Hoxa2* overexpression allele *Wnt1-Hoxa2-low*, *Wnt1-Hoxa2-medium* [16] and *Wnt1-Hoxa2-high*. *Hoxa2* knock-in loci, promoters, presence of WPRE, protein tags, IRES-EGFP and *Hoxa2* mRNA levels in PA1, as compared with endogenous levels in wild-type (wt) PA2, are indicated. (Lower rows) Comparison of hypoplastic craniofacial phenotype and homeotic transformation of proximal PA1 into PA2-like derivatives from the distinct alleles, as indicated. Skeletal elements derived from PA1 (magenta), PA2 (light green) and PA3 (dark green) cranial neural crest cells (CNCCs) are indicated. Penetrance of PA1 to PA2 repatterning phenotypes is indicated for the different alleles. dnt, dentary; f, frontal; g, gonial; gh, greater horn of hyoid bone; h, hyoid bone; hbb, body of hyoid bone; i, incus; lh, lesser horn of hyoid bone; m, malleus; mx, maxillary; n, nasal; p, pinna; pmx, premaxillary; PA, pharyngeal arch; sp, styloid process; sq, squamosal; st, stapes; tr, tympanic ring; *, duplicated structure.

These contradictory results are likely due to the different experimental approaches in distinct model organisms. Here, we investigated whether different *Hoxa2* ectopic expression levels could explain the distinct morphological phenotypes reported in the different studies. We found that differential ectopic *Hoxa2* expression levels lead to distinct craniofacial phenotypes (summary schemes in Figure 4). Namely, 60% of physiological expression levels of *Hoxa2* (*Wnt1-Hoxa2-low*) were sufficient to induce the formation of an ectopic duplicated pinna in *Hox*-negative CNCCs (Figure 2 and summary scheme in Figure 4; Minoux et al., 2013 [11]), while ectopic *Hoxa2* expression at physiological levels (100%; *Wnt1-Hoxa2-medium*) allowed for the repatterning of proximal PA1 CNCCs into a full set of PA2-like skeletal elements (summary scheme in Figure 4; [16]). This indicated that *Hox*-negative CNCCs display differential sensitivity to ectopic *Hoxa2* expression levels, and, indirectly, suggested that, in wild-type PA2 CNCCs, lower *Hoxa2* expression levels are required for pinna than middle ear and hyoid cartilage patterning. In contrast, high levels of ectopic *Hoxa2* overexpression, namely 200% of its physiological levels (*Wnt1-Hoxa2-high*) generated the most severe phenotype, with almost complete aplasia of craniofacial cartilage and bone elements, including the proximal PA1 and PA2 derivatives. This analysis indicates that the severity of hypoplasia/malformation, or aplasia, of craniofacial structures correlates with *Hoxa2* overexpression levels. Altogether, these results reconcile the discrepancies in previously published experiments and indicate that distinct subpopulations of *Hox*-negative CNCCs are differentially sensitive to ectopic levels of *Hox* expression.

Our results, together with previous studies, also indicate that the ability of ectopic *Hoxa2* to specify CNCC patterning information is position- and context-dependent. In *Hoxa2* knockout mouse fetuses, only derivatives of the proximal part of PA1, including the middle ear ossicles incus and malleus-homologous to the quadrate and articular cartilage forming the primary upper and lower jaw joint articulation in non-mammalian jawed vertebrates (the ‘hinge’ region of PA1 [32]), were duplicated into the PA2 territory, while the distal part of PA1, including the distal portion of the lower jaw cartilage, and FNP structures were not [9,10,12]. It was therefore proposed that *Hoxa2* specifies PA2 CNCCs’ identity by modifying an underlying *Hox*-negative ground (default) patterning program, shared by the proximal PA1 and PA2 CNCCs [3,4,10,12,13,17]. In our gain-of-function experiments, we observed that even when the ectopic expression of *Hoxa2* was close to its physiological expression levels (i.e., *Wnt1-Hoxa2-low* and *Wnt1-Hoxa2-medium* fetuses), only the proximal PA1 CNCC derivatives (pinna and/or middle ear skeletal elements) could acquire a PA2-like identity. In contrast, the other *Hox*-negative CNCCs’ subpopulations, including those contributing to the distal PA1 and FNP, could not be repatterned. Their development was instead impaired, with varying degrees of severity, depending on the ectopic expression levels of *Hoxa2*. The two distinct categories of phenotypes observed by *Hoxa2* ectopic expression in *Hox*-negative CNCCs (i.e., repatterning of proximal PA1 versus hypoplasia or aplasia of distal PA1 and FNP derivatives) indicate that among *Hox*-negative CNCCs, only those sharing the ground (default) patterning program with PA2 (and more posterior PAs [7]), can acquire a PA2-like identity upon ectopic expression of *Hoxa2*, at the condition that its ectopic expression levels were close to its physiological ones.

The positional identity of the *Hox*-negative CNCCs is not yet established at the premigratory stage. Indeed, *Hox*-negative premigratory CNCC progenitors can replace each other in building a whole craniofacial skeleton [33]. Because they share similar developmental potential, these progenitor cells have been proposed to behave as an ‘equivalence group’ [33]. Recently, we discovered that premigratory CNCC progenitors display a prepatterned, transcriptionally poised, repressive chromatin organization that maintains their broad developmental plasticity through migration [34]. In response to position-specific environmental signals that the CNCCs meet during or after their migration, genes encoding for key transcription factors are locally transcriptionally induced, thus establishing positional identity of each *Hox*-negative CNCCs’ subpopulations (e.g., FNP and mandibular and maxillary processes of PA1), driving the morphogenetic program to make the right structure in the right place [34]. This further suggests that the *Hox*-negative ground (default) patterning program shared by proximal PA1 and PA2 CNCCs is established in post-migratory CNCCs.

As a corollary, the ability of *Hoxa2* to repattern *Hox*-negative CNCCs would then absolutely depend on the presence of permissive local environmental signal(s). The mirror image homeotic transformation of PA2 into a subset of PA1 derivatives in *Hoxa2* knockout mutants strongly implies that (at least part of) PA1 and PA2 derived CNCCs are bi-directionally instructed by the same set of ectodermal and/or endodermal signals at the pharyngeal cleft and/or pouch between PA1 and PA2, and that their distinct morphological readouts solely depend on whether *Hoxa2* is expressed (i.e., in PA2) or not (i.e., in proximal PA1) [10,12]. Hence, among the *Hox*-negative CNCCs, only those able to respond to the same signal(s) that pattern PA2 CNCCs, i.e., the proximal PA1 CNCCs, would acquire a PA2-like identity upon ectopic expression of *Hoxa2*. In contrast, overexpression of *Hoxa2* in the other *Hox*-negative CNCCs’ subpopulations, including distal PA1 and FNP, would make them incompatible to appropriately respond to the local environmental signals that normally instruct them, causing a dramatic impairment of the development of the corresponding skeletal structures [30]. In this respect, it is interesting to note that *Wnt1-Hoxa2-low* fetuses occasionally display ectopic structures around the eye, morphologically resembling small ectopic pinnae, suggesting that signals compatible with pinna formation are also present there [11].

All three *Wnt1-Hoxa2-low*, *Wnt1-Hoxa2-medium* and *Wnt1-Hoxa2-high* mouse lines display hypoplasia of craniofacial structures. However, while hypoplasia was relatively modest in *Wnt1-Hoxa2-low* and *Wnt1-Hoxa2-medium* fetuses (Figure 2 and Figure 4), strong *Hoxa2* overexpression in *Wnt1-Hoxa2-high* fetuses resulted in almost complete aplasia of craniofacial skeletal elements, including the lack of structures derived from proximal PA1 and PA2 CNCCs (Figure 1B; see also summary schemes in Figure 4). This might be because high ectopic and constitutive *Hoxa2* expression eventually impairs chondrocyte survival and/or differentiation [29,31], even in proximal PA1 CNCCs. In this respect, *Hoxa2* expression is normally switched off in PA2 *Sox9*-positive CNCC-derived chondrogenic condensations, and constitutive overexpression of *Hoxa2* in these cells interferes with chondrogenesis [19]. Moreover, *Hox* expression must be turned off for chondrogenic and osteogenic differentiation to proceed normally in the long bones of the skeleton, while being retained in the perichondrium immediately surrounding these elements [35].

### 4.2. Hoxa5 Overexpression Cannot Homeotically Repattern CNCCs and Generates Distinct Phenotypes Compared With Hoxa2 Overexpression

In the chick, ectopic expression of *Hox* genes of the first four paralogue groups (*Hoxa2*, *Hoxa3* or *Hoxb4)* in *Hox*-negative CNCCs resulted in negative, albeit not identical, effects on the development of the craniofacial skeleton, with no homeotic phenotypes [18]. In particular, the effects of *Hoxa3* and *Hoxb4* ectopic expression were more restricted, selectively affecting the chondrogenesis of distinct CNCC derivatives [18]. Given the severity of these phenotypes, it was, however, not possible to evaluate whether, under different conditions, ectopic expression of *Hox* genes other than *Hoxa2* might also induce repatterning of PA1 into PA2-like derivatives.

Here, we compared the craniofacial phenotypes of *Wnt1-Hoxa2-high* and *Wnt1-Hoxa5* fetuses, in which *Hoxa2* and *Hoxa5* were strongly expressed in CNCCs using the same induction systems (i.e., *ROSA26* locus, *CAG* promoter, *WPRE* sequence). We found that overexpression of *Hoxa5*, a *Hox* gene normally not expressed in CNCCs giving rise to craniofacial structures, surprisingly induced milder hypoplasia of FNP and distal PA1 derivatives, as compared to *Hoxa2* overexpression. In contrast, the pharyngeal arch CNCC derivatives, including proximal PA1 and PA2–PA4 structures (i.e., middle ear, hyoid, thyroid, pinna) were notably absent in *Wnt1-Hoxa5* fetuses, thus marking a remarkable difference as compared with *Hoxa2* overexpression. Moreover, in *Wnt1-Hoxa5* fetuses there was no homeotic transformation of PA1 into PA2-like elements (summary schemes in Figure 3I). Our results confirm that, even though *Hoxa2* and *Hoxa5* overexpression levels in CNCCs were likely similar, they resulted in relatively distinct and specific phenotypes, consistent with the observations in chick embryos by Creuzet et al., 2002 [18].

Finally, it is still relatively unclear how Hox proteins select and bind to their target sequences in vivo to regulate specific downstream target genes during morphogenesis. In this respect, even though beyond the scope of this study, our experimental design might be useful as it would allow to compare the direct binding sites of different Hox transcription factors, expressed at similar levels, in mouse embryonic tissues and cell types in vivo. For instance, since CNCCs are labelled by *IRES-EGFP* in *Wnt1-Hoxa2-high* and *Wnt1-Hoxa5* embryos it is possible to isolate them by FACS, as we previously did with *Wnt1-Hoxa2-low* embryos [34]. Moreover, because both Hoxa2 and Hoxa5 are tagged by the 3XFLAG epitope, it is possible to perform ChIP-seq against these transcription factors, in comparable conditions, to identify their specific and shared direct targets in the genome. This would represent a valuable approach to understand the molecular mechanisms of *Hox* gene-mediated cell identity specification during development.

## Data Availability

Access to mouse lines and images are available upon request.

## Supplementary Materials

The following supporting information can be downloaded at: https://www.mdpi.com/article/10.3390/jdb10010009/s1, Figure S1: EGFP signal in E18.5 *Wnt1-Hoxa5* mouse fetus.
