# Supplementary material for: Different Ectopic Hoxa2 Expression Levels in Mouse Cranial Neural Crest Cells Result in Distinct Craniofacial Anomalies and Homeotic Phenotypes
Source: J Dev Biol. 2022 Jan 31;10(1):9. doi: 10.3390/jdb10010009 (PMC8883995; doi:10.3390/jdb10010009)
Supplement: Supplementary file 1 [file jdb-10-00009-s001.zip › jdb-1541458-supplementary.pdf]

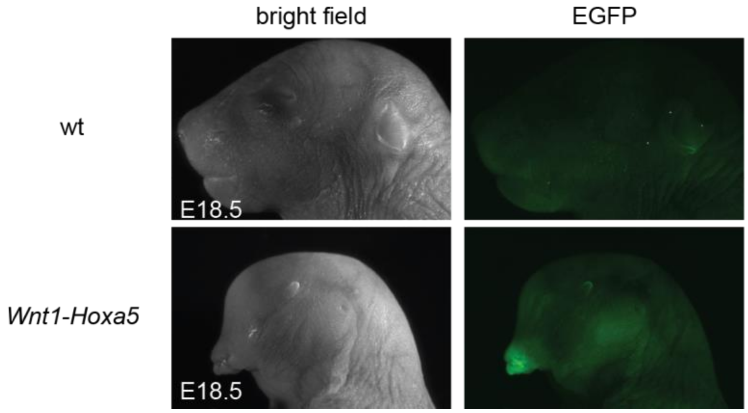

Figure S1. EGFP signal in E18.5 *Wnt1-Hoxa5* mouse fetus. E18.5 wild-type (wt, top) and *Wnt1-Hoxa5* (bottom) fetuses in bright field (left) and EGFP (right) signals; note that above-background EGFP signal is visible underneath the skin in underlying facial structures of *Wnt1-Hoxa5* specimen.
